# A Pilot Study: Dietary Energy Density is Similar between Active Women with and without Exercise-Associated Menstrual Dysfunction

**DOI:** 10.3390/nu8040230

**Published:** 2016-04-19

**Authors:** Taryn M. Hand, Stephanie Howe, Lynn Cialdella-Kam, Charlotte P. Guebels Hoffman, Melinda Manore

**Affiliations:** 1 Nutrition and Exercise Science, School of Biological and Population Health Sciences, Oregon State University, Corvallis, OR 97331, USA; tarynmhand@gmail.com (T.M.H.); Stephaniemariehowe@gmail.com (S.H.); 2 Department of Nutrition, School of Medicine, Case Western Reserve University, Cleveland, OH 44106, USA; lynn.kam@case.edu; 3 Tahoe Forest Hospital, Tahoe City, CA 96146, USA; choffman@tfhd.com

**Keywords:** energy availability, energy balance, exercise energy expenditure, amenorrhea, female athletes

## Abstract

Low energy availability (EA) (e.g., insufficient energy intake (EI) to match energy needs, including exercise energy expenditure) has been identified as a primary contributor to exercise-associated menstrual dysfunction (ExMD) in active women. For health reasons, active women may self-select diets lower in energy density (ED, kcal/g), which can inadvertently contribute to inadequate EI. Using data from two studies, we compared the ED of active women with ExMD (*n* = 9; 24 ± 6 years) to eumenorrheic (EU) active controls (EU: *n* = 18, 27 ± 6 years). ED was calculated from 6 to 7 days weighted food records using two methods: with/without beverages. ANOVA and Wilcoxon Rank-Sum were used to test group differences. ED was not different between groups, but there was a trend toward a lower median ED (10%) (*p* = 0.049 unadjusted; *p* = 0.098 adjusted) in the ExMD-group (Method 1—all beverages: ExMD = 1.01 kcal/g (range = 0.52–1.41), EU = 1.22 kcal/g (range = 0.72–1.72); Method 2—without beverages: ExMD = 1.51 kcal/g (range = 1.26–2.06), EU = 1.69 kcal/g (range = 1.42–2.54)). This lower ED represents a 9% decrease (~219 kcal/day) in EI (ExMD = 2237 ± 378 kcal/day; EU = 2456 ± 470 kcal/day; *p* > 0.05). EI and macro/micronutrient intakes were similar for groups. In the ExMD-group, low ED could contribute to lower EI and EA. Future research should examine the interaction of ED and exercise on appetite, EI, and EA in active women, especially those with ExMD.

## 1. Introduction

Exercise-associated menstrual dysfunction (ExMD) affects between 6% and 79% of active women, with ~50% of female athletes exhibiting some form of ExMD [1,2]. Women participating in sports that emphasize leanness and/or require revealing clothing are especially at risk [1,2]. To maintain a lean build, female athletes frequently restrict energy intake (EI), leading to poor energy and nutrient intakes, suppression of reproductive function, and poor bone health (e.g., Female Athlete Triad) [3]. Additionally, when athletes have chronic energy deficit/low-energy availability (EA) other health issues can occur, including impaired immunity, depressed protein synthesis, slower injury recovery and increased risk factors for cardiovascular disease [4]. The International Olympic Committee has identified this cascade of events as relative energy deficiency in sport (RED-S) [4], which includes the health issues associated with the Triad. For many active women, RED-S can be attributed to a conscious effort to restrict EI or inadvertently not consuming enough energy, such as eating a low-energy dense (low-ED) diet.

Energy density (ED) is defined as energy (kcal) per gram weight (g) of food, thus, low-ED diets have a high volume of food with relatively low energy content. The ED of the diet can be classified as very low (<0.6 kcal/g), low (0.6–1.5 kcal/g), medium (1.5–4.0 kcal/g) or high (>4.0 kcal/g) [5]. Low-ED diets have been effectively used for weight loss in overweight/obese groups [6,7,8], which aims to replace high-ED foods (e.g., chips, sweets, nuts, cheese) with low-ED foods high in water, volume and fiber, resulting in an increased sense of fullness after eating. Low-ED foods include whole fruits and vegetables, whole grains (e.g., wet grains such a brown rice or oatmeal) and beans/legumes, and low-fat meat and dairy foods. Health conscious active women frequently self-select low-ED diets, which are recommended for good health [9], but they may satiate before providing enough energy to cover energy expenditure. Thus, this dietary approach may place highly active women at risk for RED-S and low-EA [3,4,10].

To date, only two studies have examined the relationship between dietary ED and ExMD in active women. Reed *et al.*, found that ED was significantly lower in active women with ExMD (22% body fat; ED = 0.77 kcal/g) than EU, ovulatory controls (25% body fat; ED = 1.06 kcal/g) [11]. However, ED was only significantly different between groups when non-caloric beverages (e.g., plain coffee, unsweetened tea, diet soda) were included in the ED calculation. Including non-caloric beverages adds weight to the diet but no energy, thus, potentially underestimating dietary ED. Melin *et al.*, found that dietary ED was similar between female endurance athletes with low EA (19.4% body fat ; ED = 1.36 kcal/g) and functional hypothalamic amenorrhea (FHA) (18.6% body fat; ED = 1.36 kcal/g); ED was calculated with all foods/no beverages [10].

The purpose of this pilot study, using data from two different research studies, was to determine if dietary ED differed in active women with/without ExMD using two different measures of ED (e.g., with/without beverages). Results would help determine if a larger research study was warranted. We hypothesize that active women with ExMD would consume low-ED diets and have higher dietary fiber intakes than EU active controls when non-caloric beverages were excluded from the calculation. A secondary question examined whether active women with high sport product (SP) intake (≥5% EI) had higher ED diets than those with low-SP use (≤5% EI).

## 2. Materials and Methods

Baseline data from two studies using female athletes were combined for this pilot study. Studies recruited recreational/competitive female athletes (*n* = 33, 18–40 years) who self-reported either regular menstrual function (eumenorrheic, EU) or had ExMD. Inclusion criteria included performing endurance exercise (>5 day/week, 30–60 min/session), not pregnant or lactating, weight stable (<2 kg weight change in past 6-months), no reported current eating disorder, and a VO_2MAX_ > 40 mL/kg/min. Studies were approved by the University Institutional Review Board and informed consent obtained.

### 2.1. Anthropometrics and Aerobic Capacity

Wearing running clothes and no shoes, height and weight were measured with a stadiometer to the nearest centimeter and a standardized balance scale to the nearest 0.1 kg, respectively. For body composition assessments, Study 1 used dual x-ray absorptiometry (DXA) (GE Lunar Prodigy QDR 4500A; Hologic, Waltham, MA, USA) with the same trained technician; Study 2 measured 7-site skinfolds using the same trained technician and applied the Jackson and Pollock equation for females [12].

Maximal aerobic capacity (VO_2MAX_) was measured on a treadmill (Max-1 PhysioDyne Instruments Corp., Quogue, NY, USA) with a heart rate monitor (Polar Electro, Inc., Lake Success, NY, USA) and ratings of perceived exertion recorded every minute. Once familiarized with the treadmill, subjects had a 4-min warm-up, then speed or incline were increased every minute until exhaustion was reached (~12-min); the cool-down period continued until heart rate returned to warm-up level. 

### 2.2. Diet and Physical Activity Assessment

Energy and dietary intakes and physical activity (PA) were assessed using 6–7-day weighed diet records and PA logs. After instruction on using food scales and logs, subjects recorded all food/beverages and 24-h PA each day. A Registered Dietitian (RD) and Certified Specialist in Sport Dietetics (CSSD) reviewed all food/PA logs for clarification. Food Processor SQL (Version 10.13, 2014; ESHA Research, Salem, OR, USA.) was used for energy and nutrient intake and energy expenditure analysis. Foods not included in the database were entered manually based on recipes and food labels. Under-reporters were identified and excluded using the Goldberg *et al.*, [13] equation (EI <1.3 × predicted resting metabolic rate (RMR)) [13]. Study 1 measured RMR using indirect calorimetry as described by Guebels *et al.*, [14] and estimated using Harris-Benedict equation [15]; Study 2 estimated RMR using the Harris-Benedict equation [15,16]. Any PA with a metabolic equivalent of task (MET) level >4 was considered exercise and reported in Table 1. 

### 2.3. Energy Density Calculation

Dietary ED (kcal/g) was calculated with/without beverages using two methods. Method 1 included all beverages except for water, included energy containing beverages such as juice, soda, milk, coffee, tea and alcohol. Method 2 included only food and liquid meal-replacement beverages (e.g., smoothies or shakes) and excluded all non-caloric/caloric (e.g., alcohol, juice, milk) beverages. Energy-containing beverages are excluded because they falsely decrease ED and do not reflect the individual’s actual dietary ED [17,18]. Liquid meal replacement beverages/liquids added to smoothies were included because they satiate and contribute to the dietary macronutrient composition [17]. ED was calculated for each day then averaged. 

### 2.4. Sport Food Intake

We determined the ED of high/low SP users. SPs included energy bars, gels and chews, recovery beverages and protein powder. The median SP use was 5% of EI, thus, high-SP users (*n* = 9) were defined as consuming >5% kcal/day from SPs, whereas low-SP users (*n* = 18) consumed ≤5% kcal/day from SP. We also subdivided SP into sport beverages (SB, e.g., protein powder drinks, recovery, electrolyte replacement beverages) and non-SB (e.g., gels, chews, energy bars) and determined their contribution to EI for each individual. 

### 2.5. Menstrual Status

Subjects were classified as either ExMD or EU based on ovulation and blood reproductive hormone assessments (Study 1) or self-report (Study 2). Menstrual status details were collected via questionnaire/interview. Subjects were classified as EU if they reported regular menstrual cycles consistently for ≥6-months and ExMD if they reported no menstrual cycles for the past 3 months or longer and no non-ExMD. In Study 1 oral contraceptive (OC) use was an exclusion criteria, while Study 2 allowed OC use if not for menstrual regulation (*n* = 3, EU = 3 for birth control). Duration and purpose for OC use and other reproductive function issues were documented. All subjects in the EU group self-reported regular menstrual function prior to OC use and, thus, were included with the EU group.

### 2.6. Statistical Analysis

Power calculations were used to determine the sample size required to detect a significant difference in ED of 0.35 kcal/g [6]. A sample size of 24/group is required to achieve a power of 80% (α = 0.05). Reed *et al.*, had similar participants and reported significant differences in ED with a sample size of 12–13/group [11]. The present pilot study was a convenience sample that included data from two separate studies, each with their own power calculations.

Data are presented as mean ± standard deviation (SD). To determine if groups differed in ED (kcal/g) and fiber intake (g/day), one-sided Wilcoxon Rank-Sum tests were used. For ED, we also examined differences between groups by calculating an effect size (ES) using the Cohen’s *d* statistics and confidence intervals (CI) determined. Group comparisons for the remaining variables were made using one-way analysis of variance (ANOVA). For all comparisons, equal variance assumption was evaluated using Levene *F* test. Statistical significance was set at *p* < 0.05 and the False Discovery Rate (FDR) test used to correct for multiple comparisons. Statistical analysis was done with JMP 11.1.1 (SAS Institute Inc., Cary, NC, USA).

## 3. Results

Overall, 33 subjects completed the study (ExMD = 9; EU = 24), but six EU subjects were identified as under-reporters and excluded; thus, 27 subjects (ExMD = 9; EU = 18) were included in the final analyses. There were no group differences for demographic and anthropometric data (See Table 1) and time spent in exercise (min/day, >4 METS = 72–82 min/day or 8.5–9.5 h/week).

### 3.1. Energy and Nutrient Intake

Energy and nutrient intake data are presented in Table 2. There were no differences between groups for energy and macronutrient intake expressed as either g/kg or percentage of EI and alcohol consumption was low (2%–4% of EI). The current Adequate Intake (AI) for fiber is >25 g/day [19] or 14 g/1000 kcal; the proportion of individuals in each group meeting these recommendations were similar (ExMD = 67%, EU = 72%; ExMD = 22%, EU = 27%, respectively).

### 3.2. Energy Density

**Figure 1:**
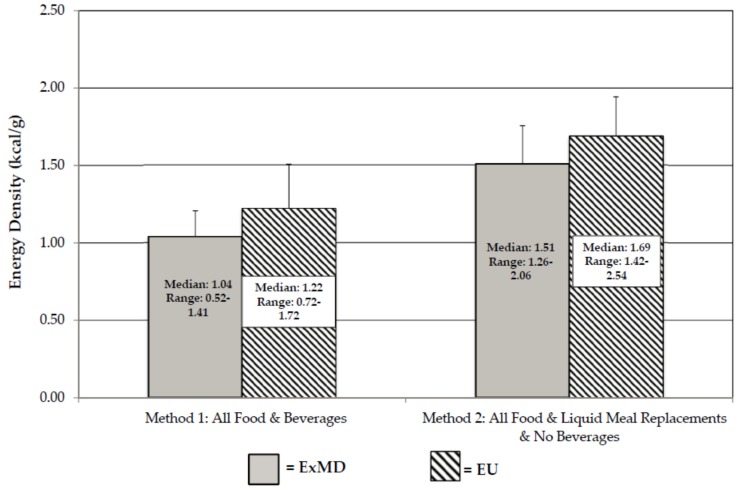
Energy Density of Exercise-Associated Menstrual Dysfunction (ExMD) and Eumenorrheic (EU) Active Women. Note: Adjusted p-values: Method 1 *p* = 0.285; Method 2 *p* = 0.098.

Energy density data are presented in Figure 1. Regardless of the ED method used, there were no group differences in ED. Using Method 2, where all beverages were eliminated from the analysis, there was a trend for lower ED in the ExMD (median ED = 1.51, range: 1.26–2.06 kcal/g) *vs.* EU group (median ED = 1.69, range: 1.42–2.54 kcal/g) (*p* = 0.049 1-sided unadjusted; *p* = 0.098 adjusted). Using Method 1, 100% of the ExMD *vs.* 67% of EU consumed a low-ED diet (0.6–1.5 kcal/g), while using Method 2, 44% of the ExMD *vs.* 11% of the EU consumed a low-ED diet. Overall, the median dietary ED was 10%–12% lower in the ExMD *vs.* EU group. When Cohen’s *d* statistic was used to calculate ES between the groups, results were similar as above. For Method 1 (beverages included) the ES was 0.59 (indicating a medium ES; mean ± SD and CI: ExMD = 1.12 ± 0.18 kcal/g; CI = 0.99, 1.25, and EU = 1.28 ± 0.28 kcal/g; CI = 1.13, 1.42). For Method 2 (no beverages) the ES was 0.70 (indicating medium ES; mean ± SD and CI: ExMD = 1.57 ± 0.25 kcal/g, CI = 1.3, 1.77, and EU = 1.75 ± 0.26 kcal/g, CI = 1.63, 1.88). Using the mean ED for each group, the ED was 10%–12% lower in the ExMD *vs.* EU, similar to what was observed when the differences were calculated using the median.

### 3.3 Sport Food Intake

Sport foods (e.g., bars, gels) are typically high-ED foods; thus, we examined their use in our participants. Overall, there were no differences in ED between low-SP (*n* = 18; 1.65 ± 0.19 kcal/g) and high-SP (*n* = 9; 1.78 ± 0.36 kcal/g) users. Based on median dietary ED values, both groups could be classified as consuming medium-ED diets (1.5–4.0 kcal/g). When SP users were grouped by menstrual status, EI from SPs was similar between groups (ExMD, *n* = 9; 3.0% ± 3.8%; EU, *n* = 18; 4.4% ± 4.6%). Overall, total EI from SBs was low in both groups at <3% of EI (ExMD = 2.5% ± 3.2% *vs.* EU = 0.6% ± 1.3%); whereas total EI from SFs was slightly higher (EU = 3.8% ± 4.2% *vs.* ExMD = 0.6% ± 1.0%).

## 4. Discussion

Regardless of method used, we found no differences in dietary ED in active women classified with/without ExMD. To date only two studies have examined ED in active women [10,11]. Our data do not support the findings of Reed *et al.*, [11] when non-caloric beverages are included in the ED calculations; they report significant differences between ExMD and EU groups. However, when under-reporters were excluded group differences disappeared. Including non-caloric beverages [17] and under-reporters in the calculation artificially lowers ED. We eliminated under-reporters and used the most universally accepted method for calculating ED, which eliminates non-caloric beverages. In addition, our mean EIs (EU = 2456 kcal/day, ExMD = 2237 kcal/day) were similar to those typically found in weight stable EU active women [20,21,22] Conversely, Reed *et al.*, [11] reported EIs ranging from 725 to 2567 kcal/day (ExMD) and 1284 to 2852 kcal/day (EU). They also reported that their active women expended 300–400 kcal/day in exercise, yet some consumed only 725–1284 kcal/day. This level of EI is below estimated RMR and would not be consistent with weight stability. 

Melin *et al.*, [10] compared the ED of female endurance athletes classified as optimal EA, low-EA, EU or FHA. ED was calculated using a “food-group approach” instead of individual foods and three different methods (all food/no beverage, all food/energy beverages, and all food/all beverages including water). When all beverages were eliminated from the ED calculation, ED and fat intake were similar between low-EA and FHA groups, but lower compared to athletes with optimal EA. They concluded that this difference could lead to low-EA athletes resulting in FHA. Conversely, when calorie-containing beverages were included there were no differences in ED between groups. Thus, it is difficult to compare these data to ours due to differences in ED calculations; however, the same trends were seen with athletes with ExMD having lower ED values.

We found no difference in the contribution of SPs to total EI between our groups. Reed *et al.*, also found that the energy contribution of SB and SF (% of energy) was similar between NCAA Division I female soccer players classified with low or high EA [23]. However, they report a higher use of SB (6%–7% of energy) and sport foods (2%–3% of energy) in their active women than we observed (3%–4% of energy) for all SPs combined. Surprisingly, our highly active endurance trained women were not high SP or SB users.

Carbohydrate recommendations for active, endurance trained women range from 5 to 7 g/kg/day during moderate training periods and up to 10 g/kg/day during intense training periods [24,25]; our subjects were not meeting this recommendation (ExMD = 5.0 ± 1.2 g/kg, EU = 4.9 ± 1.3 g/kg). It is especially important for women who are actively competing to consume the higher end of this range to help glycogen replenishment during intense training sessions and competitions. Mean protein intakes (g/kg) were within recommended ranges for active, endurance trained women (1.2–1.4 g/kg) (ExMD = 1.4 ± 0.2 g/kg, EU = 1.6 ± 0.4 g/kg) [24,25], with only one participant consuming less than the RDA requirement for protein (0.8 g/kg).

Although reported exercise time (min/week) was similar between the groups, the ExMD group consumed ~219 kcal/day less (9% lower EI) and had a 10% low-ED diet. These differences in EI and ED are consistent with dietary interventions aimed at weight loss [6,11,26,27,28]. Taken together, these studies show that for each percentage decrease in ED, dietary EI is reduced by a similar percentage [6,8,11,26]. The ED of the typical American diet is ~1.7–1.8 kcal/g, whereas weight loss interventions aim to reduce ED to 1.3–1.4 kcal/g [6,8,18,26]. The dietary ED of the ExMD group was similar to the ED of diets recommended for weight loss, while the ED of the EU group is more reflective of the typical American diet.

Contrary to our hypothesis, mean dietary fiber intake did not differ between the groups (ExMD = 11.6 g/1000 kcal; EU = 12.9 g/1000 kcal) and a similar percentage of subjects (22% ExMD; 27% EU) exceeded the AI for fiber (>14 g/1000 kcal/day). Low-ED diets are typically high in fiber rich foods that can increase satiety. Our data support that of Reed *et al.*, [11] when fiber data are expressed as g/kg (ExMD = 0.6 ± 0.3; EU = 0.4 ± 0.2; *p* = 0.193); however, when fiber data are expressed as g/1000 kcal/day (ExMD = 17.6 ± 6.4; EU = 12.1 ± 4.2; *p* = 0.018) they found significant differences between ExMD and EU groups, while we found no differences. Melin *et al.*, found lower fiber intake in EU (11.6 g/1000 kcal) *vs.* FHA (17.1 g/1000 kcal) [10]. Our fiber intakes for EU athletes are consistent with the fiber intakes reported for EU athletes by Reed *et al.*, and Melin *et al.* [10,11].

Our findings and those of others [10,11] highlight the discrepancies associated with measuring and reporting ED in active individuals. Since athletes use SB and shakes, the inclusion/exclusion of these beverages in the ED calculation needs to be standardized. The exclusion of beverages from these calculations is done because beverages add significant weight to the diet but less satiating than whole foods. Houchins and colleagues reported that both lean and obese subjects found whole fruit more satiating than fruit juice [27]. Beverages that contain added sugar, fat and protein (e.g., sport shakes, liquid meal replacement drinks) have higher ratings of satiety compared to isocaloric sugar-only drinks (e.g., soda, most sport drinks) [28]. Similarly, DellaValle and colleagues found that EI increased when caloric beverages were added to a meal without a subsequent increase in hunger or satiety ratings [29]. Thus, the inclusion of beverages, especially non-caloric beverages, can disproportionately lower ED and not reflective of the actual dietary ED. We conclude that liquid meal replacement beverages (*i.e*., macronutrient composition similar to whole food, such as smoothies/sport shakes) should be included in the calculation of ED because they affect appetite similar to whole food.

### 4.1. Strengths and Limitations

Assessing the dietary ED of active women is a new area of research, with only three studies to date including this study [10,11]. These studies all used different methods for calculating ED, making comparisons difficult and underscoring the need to standardize this assessment method in active individuals. Our results do not support the primary outcome of Reed *et al.* [11], who reported a significant difference in dietary ED between active women with/without ExMD.

We used highly active and elite endurance athletes who may face added pressure to be thin for performance or aesthetic reasons, which is a unique feature and strength of our study. This group is often under-represented in research due to the difficulty of finding subjects willing to interrupt their training cycles to participate. Thus, this research describes the dietary behaviors of highly active women.

Self-reported food records are prone to under-reporting; however, participants were carefully trained by a RD to weigh and record foods and we screened and eliminated for under-reporters. Unfortunately, due to the research protocol for one study, we were unable to screen for eating disorders using a validated screening measure and estimate EA. However, all subjects were thoroughly interviewed by a RD trained in sport nutrition (CSSD credential) to assess for potential disordered eating behaviors.

Ideally, menstrual status is confirmed by using laboratory tests rather than self-report. Without these laboratory data, it is impossible to tell whether sub-clinical menstrual disturbances exist [30,31]. Study 2 design did not allow for assessment of menstrual function using laboratory data; however, we carefully confirmed classification of menstrual status based on frequency of menses and menstrual history. Subjects using OCs were questioned regarding reasons for use (e.g., birth control, resumption of menses). We only included those women using OCs if they reported regular menstrual cycles prior to OC use and had no history of ExMD. No participants self-reported a current medically diagnosed eating disorder, thus we confidently eliminated under-reporters from the analysis. 

Additionally, this pilot study used data from two studies to determine if there was the potential for ED to be different between these two groups of active women. The original studies were not specifically designed to specifically address this research questions. Once under-reporters were eliminated, the sample size appeared to be under-powered to determine significant differences between the groups. Because of the difficulty in recruiting active women with verified ExMD, we used a convenience sample, but this limited our ability to achieve a well-balanced group size that was adequately powered. While we did see a 10% difference in ED, this difference was not statistically significant, yet it could be one contributor in the lower EI and EA observed in active women with ExMD. More research specifically targeted at examining this question is warranted.

### 4.2. Application

This study adds to the growing body of research examining ED in the diets of active women in lean-build sports. While ED was not significantly different between groups, the ED of the ExMD group was similar to that used in weight loss interventions. This information can be helpful to sport dietitians and other health professionals who make dietary recommendations to active women. For some active women, consuming a low-ED diet may not provide enough energy and nutrients to meet the increased energy demands of exercise, thus, leading to energy deficiency and increased risk for ExMD. For example, 44% of our ExMD female athletes consumed a low-ED diet (Method 2) compared to only 11% in the EU group. When low-ED diets are combined with the appetite blunting effect of high intensity exercise [32,33,34,35], the results may be inadequate EI from whole foods alone. Thus, exploring these two factors in the lifestyles of active women with ExMD could lead to better dietary strategies for reversing ExMD. These individuals may need to increase the consumption of healthy, high-ED foods (e.g., nuts/nut butters, avocado, oils, hummus, granola, and cheese). They may also need to increase the use of SPs during high intensity exercise training sessions (e.g., a sport bar or shake) to boost overall daily EI. To ensure energy needs are met during periods of high energy expenditure, highly active women, especially those with ExMD, should include some moderate-high ED foods as part of their regular training and recovery dietary plan.

## 5. Conclusions

Regardless of method used, we found no differences in dietary ED in active women classified with/without ExMD. However, the median ED of the ExMD group was similar to the ED of diets used in weight loss interventions with overweight or obese individuals. When low-ED diets are combined with the appetite blunting effect of high intensity exercise active females may not have adequate energy to support menstrual function. In the ExMD-group, low ED could contribute to lower EI and EA. Future research should carefully measure the ED of food following a standardized protocol so studies can be compared, assure that diet is measured over the training period of the athletes, and under-reporters are eliminated. 

## Figures and Tables

**Table 1:** Demographic Characteristics of Exercise-Associated Menstrual Dysfunction (ExMD) and Eumenorrheic (EU) Groups ^1^.

Description	ExMD (*n* = 9)	EU (*n* = 18)	*p*
Age (year)	24.3 ± 6.0	27.6 ± 6.0	0.191
Age at Menarche (year)	13.4 ± 1.9	13.1 ± 1.2	--
Gynecological Age (year) ^2^	10.9 ± 6.8	14.5 ± 5.6	--
Height (cm)	165.6 ± 6.6	168.4 ± 5.5	0.246
Weight (kg)	60.1 ± 10.1	63.5 ± 8.6	0.366
Body Mass Index (BMI) (kg/m^2^)	21.8 ± 2.8	22.3 ± 2.5	--
Fat Free Mass (FFM) (kg)	47.3 ± 5.7	51.1 ± 4.5	0.066
Body Fat (BF) (%)	20.7 ± 6.0	19.0 ± 5.7	0.479
VO_2MAX_ (mL/kg/min)	49.3 ± 5.5	53.0 ± 5.2	0.104
Exercise > 4.0 METs (min/day) ^3^	82 ± 36	72 ± 48	--

^1^ All data are reported as means ± standard deviation; ^2^ Gynecological age is the time since onset of menarche, and is computed as current age subtracted by age at menarche; ^3^ MET= Metabolic Equivalent of Task defined as 1 kcal/kg/h.

**Table 2:** Active women classified as Exercise-Associated Menstrual Dysfunction (ExMD) or Eumenorrheic (EU) Active Controls: Energy and Nutrient Intakes from 6 to 7 day Weighed Food Records ^1^.

Description	Units	ExMD (*n* = 9)	EU (*n* = 18)	*p*
Total Energy	kcals/day	2237 ± 378	2456 ± 470	0.316
	kcal/kg	37.8 ± 6.7	39.1 ± 7.9	--
Protein	g/kg	1.4 ± 0.2	1.6 ± 0.4	0.269
	% energy	15.1 ± 3.2	16.5 ± 3.3	--
	g/day	83.7 ± 18.9	99.8 ± 24.4	--
Carbohydrate	g/kg	5.0 ± 1.2	4.9 ± 1.3	0.758
	% energy	52.8 ± 5.9	49.9 ± 6.6	--
	g/day	296 ± 64	309 ± 79	--
Fat	g/kg	1.3 ± 0.3	1.4 ± 0.3	0.269
	% energy	30.1 ± 3.7	33.3 ± 5.3	--
	g/day	74.8 ± 15.6	89.8 ± 21.1	--
Alcohol	g/kg	0.2 ± 0.2	0.1 ± 0.2	--
	% energy	3.8 ± 3.6	2.0 ± 3.1	--
	g/day	11.4 ± 12.9	7.6 ± 10.4	--
Fiber	g/1000 kcal/day	11.6 ± 3.1	12.9 ± 3.3	0.220 ^2^
	g/day	28.5 ± 8.7	32.2 ± 11.3	--
Caffeine	mg/day	154 ± 113	106 ± 106	--
Calcium	mg/day	1268 ± 556	1201 ± 407	--
Magnesium	mg/day	289 ± 107	355 ± 176	--
Iron	mg/day	27.2 ± 15.3	21.8 ± 8.2	--
Folate	mcg/day	516 ± 441	495 ± 288	--
Vitamin B_6_	mg/day	3.8 ± 2.7	3.0 ± 2.0	--
Vitamin B_12_	mcg/day	12.3 ± 23.7	6.8 ± 4.4	--
Vitamin D	IU/day	360 ± 306	246 ± 267	--

^1^ All data are reported as means ± standard deviation; ^2^ Represents adjusted, one-sided for fiber (g/1000 kcal/day), other p-values are two-sided.
